# The Role of Double J Stenting in Nonobstructive Acute Pyelonephritis With Acute Kidney Injury

**DOI:** 10.7759/cureus.98501

**Published:** 2025-12-05

**Authors:** Raniya Palliyedath, Ram Prabahar M, Jayanivash Jayam, Sathiyan Sivanandam, Muthu Veeramani, Nivedita C

**Affiliations:** 1 Internal Medicine, SIMS Hospitals, Chennai, IND; 2 Nephrology, SIMS Hospitals, Chennai, IND; 3 Urology, SIMS Hospitals, Chennai, IND

**Keywords:** acute pyelonephritis, aki, conservative treatment, dj stent, minimally invasive

## Abstract

Background and objective

There has been a paradigm shift in the clinical manifestations and renal outcomes of patients with nonobstructive acute pyelonephritis (NO-APN), with acute kidney injury (AKI) being a common complication. While the standard of care remains parenteral antibiotics, minimally invasive urologic procedures is still uncertain. This study aimed to assess the effectiveness of double J (DJ) stenting in patients with NO-APN with AKI in promoting renal recovery, assessed by improvements in creatinine and inflammatory markers.

Methods

This retrospective study examined the renal outcomes of patients with NO-APN and AKI admitted to a tertiary care hospital in Chennai, India. Patients were divided into two cohorts according to the treatment received: conservative management (Group A) and DJ stenting (Group B). Treatment outcomes were assessed in terms of renal recovery, sepsis resolution, and length of hospital stay. Additionally, a pre-specified subgroup analysis was conducted comparing Kidney Disease: Improving Global Outcomes (KDIGO) Stage III AKI patients between Group A and Group B.

Results

There was no statistically significant difference between the conservative (Group A) and intervention (Group B) patients in terms of overall renal outcomes. However, in the subgroup analysis of Stage III AKI patients, those who underwent DJ stenting (Group B) demonstrated better treatment outcomes than the conservative group. There was a statistically significant reduction in mean total leukocyte count (TLC) (from 14,937.9 ± 5,275.5 cells/mm³ at admission to 11,656.3 ± 4,936.1 cells/mm³ at discharge, *p* = 0.03) and serum creatinine (from 3.46 ± 2.50 mg/dL at admission to 2.19 ± 1.93 mg/dL at discharge, *p* = 0.05) in Group B, while the duration of hospitalization was similar between groups.

Conclusions

DJ stenting can be considered a minimally invasive adjunct to intravenous antibiotics in cases of NO-APN with Stage III AKI.

## Introduction

A paradigm shift has emerged in the clinical presentation and renal outcomes of patients with acute pyelonephritis (APN) over the past two decades. Historically, acute kidney injury (AKI) secondary to APN was uncommon and was reported only in complicated cases such as emphysematous pyelonephritis and in those with anatomically abnormal or obstructed urinary tracts. However, recent studies report that the incidence of AKI in APN ranges from 60 to 80% [1,2]. Data from the Indian Society of Nephrology-AKI registry indicate that sepsis is the leading cause of community-acquired AKI, with urosepsis responsible for 20% of cases [3]. A study from a tertiary care center in North India reported an AKI incidence of 83%, with half requiring dialysis support [2]. However, data on nonobstructive APN (NO-APN) causing AKI remain scarce, with reported incidence ranging from 12 to 20%. The mechanism of AKI in NO-APN is multifactorial, involving cytokine-mediated injury secondary to sepsis and direct tubular injury [4].

According to the Infectious Diseases Society of America (IDSA) guidelines, the standard of care for NO-APN with AKI is conservative management with parenteral antibiotics for one to two weeks, while minimally invasive urological intervention is considered only in cases with poor prognostic factors or progressive disease despite antibiotics [5]. However, the benefit of cystoscopy-guided double J (DJ) stenting in such cases remains under-reported. The absence of a standardized protocol contributes to wide variation and uncertainty regarding its role. This study aimed to assess the effectiveness of DJ stenting in patients with NO-APN progressing to AKI.

## Materials and methods

Study design and setting

This retrospective, hospital-based observational study was conducted in the Department of Nephrology, SRM Institute of Medical Sciences, Chennai, Tamil Nadu, from March 2022 to December 2023. The objective was to evaluate the effectiveness of DJ stenting versus conservative antibiotic therapy in managing NO-APN complicated by AKI.

Inclusion and exclusion criteria

Patients aged >18 years diagnosed with NO-APN and AKI were included. Exclusion criteria comprised obstructive or emphysematous pyelonephritis, hydronephrosis, perinephric abscess, pyonephrosis, cases requiring percutaneous nephrostomy or drainage, APN without AKI, and patients with pre-existing CKD. These criteria minimized bias from urinary obstruction or severe coexisting pathology.

Sample size and sampling technique

Based on prior data indicating a 14.5% AKI incidence in NO-APN, and assuming a 10% margin of error with 5% precision, the minimum required sample was 90. Owing to the rarity of NO-APN with AKI, purposive sampling identified 108 eligible cases.

Data collection and variables

Clinical, demographic, and laboratory parameters were obtained from hospital records, including age, sex, comorbidities, clinical features, urinalysis, urine and blood cultures, and anthropometrics. Treatment modality was recorded as follows:

Group A: conservative antibiotic therapy

Group B: DJ stenting plus antibiotics

Outcomes assessed were serum creatinine and total leukocyte count (TLC) at admission and discharge. A pre-specified subgroup analysis was employed to compare Stage III AKI patients managed conservatively (Group A) with those undergoing DJ stenting (Group B). AKI staging followed the Kidney Disease: Improving Global Outcomes (KDIGO) Clinical Practice Guidelines for Acute Kidney Injury [4], and management adhered to the IDSA 2025 Clinical Practice Guideline for Complicated Urinary Tract Infections [5].

The decision to apply DJ stenting was made by treating physicians, and the most common indication for DJ stenting in Group B was progressive/worsening AKI or persistent fever after 48 hours of appropriate intravenous antibiotics. All these patients underwent diagnostic cystoscopy under general anesthesia, followed by fluoroscopy-assisted over-the-wire placement of DJ stents.

Identification and control of confounding factors

Potential confounders, including comorbidity burden, baseline renal function, and infection severity, were identified at baseline. Only confirmed NO-APN cases with AKI lacking urinary obstruction or advanced infection (per exclusion criteria) were analyzed to reduce bias.

Statistical analysis

Data were analyzed using SPSS v22.0 (IBM Corp., Armonk, NY). Continuous variables are presented as mean ± standard deviation (SD) and categorical variables as frequency (%). Proportions were compared using the Z-test, and intra-group differences using paired t-tests. A p-value < 0.05 was considered statistically significant.

Ethical considerations

Ethical approval was obtained from the Institutional Ethics Committee, SIMS (Ref. SIMS IEC/Others/02/2022; dated March 14, 2022). Patient data were anonymized, and all procedures complied with institutional and international ethical standards. No questionnaires or proprietary scales were employed. All clinical classifications (KDIGO and IDSA) are publicly accessible and appropriately cited.

## Results

**Figure 1:**
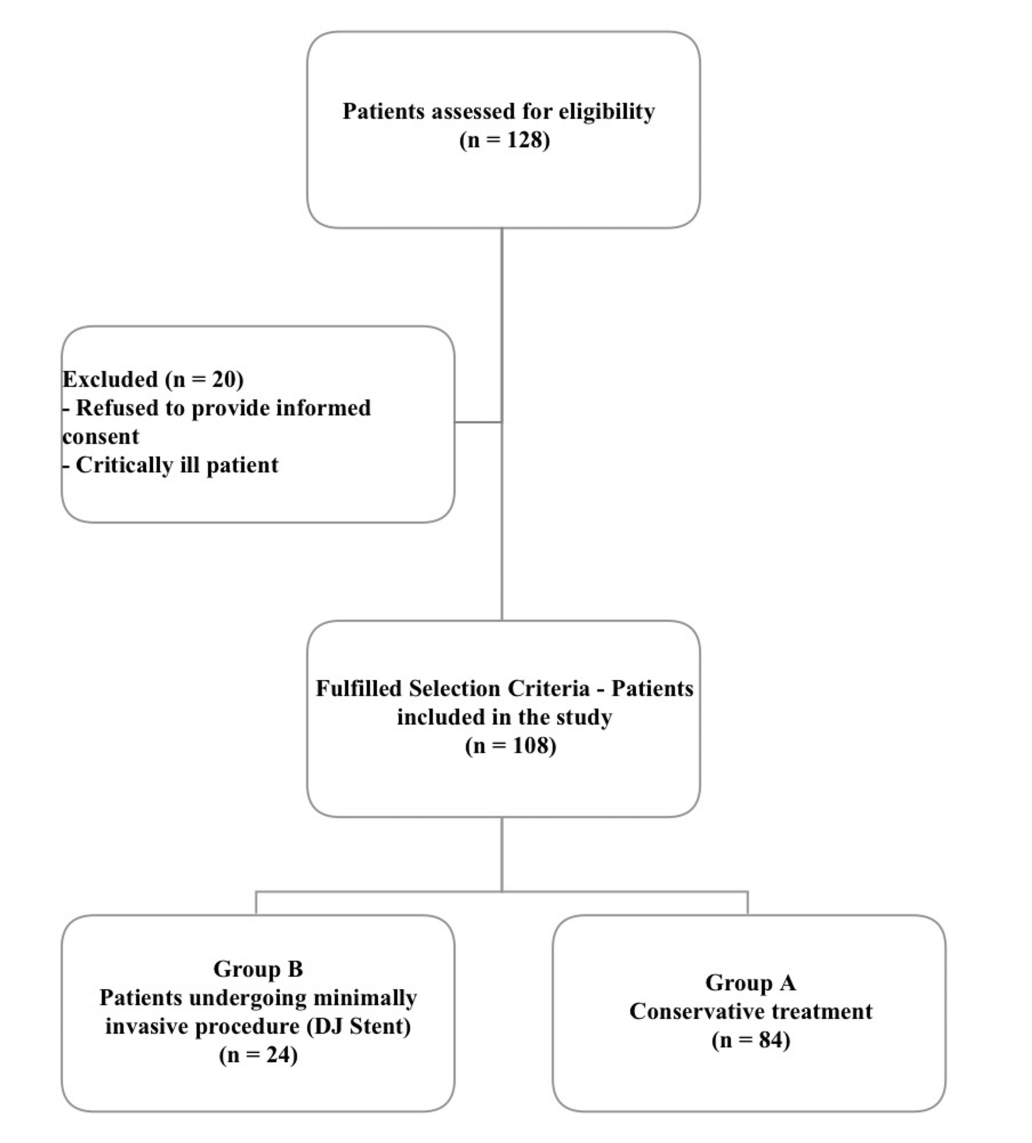
CONSORT flowchart of recruited participants CONSORT: Consolidated Standards of Reporting Trials

In this study, 128 patients with NO-APN and AKI were initially assessed for recruitment. Of these, five patients did not provide informed consent, and 15 critically ill patients were excluded to avert selection bias. The remaining 108 patients met the inclusion criteria: 84 in Group A (conservative treatment) and 24 in Group B (DJ stenting). For subgroup analysis, 20 patients from Group A with Stage III AKI were compared separately with Group B. Details of recruitment are depicted in the CONSORT flow chart (Figure 1).

The demographic and clinical characteristics of the enrolled patients are summarized in Table 1. Statistical differences were assessed between the two treatment groups. The mean age in Group A was 61.4 ± 16.5 years, and that in Group B was 70.4 ± 8.2 years. Hypertension was more prevalent in Group A (11.9%) than in Group B (4.2%) (p < 0.01). Diabetes was equally distributed, but Group B showed a significantly higher mean HbA1c (9.3 ± 2.1% vs. 8.4 ± 1.7 %, p = 0.03). A significant history of urosepsis was observed in all Group B patients and was more frequent than in Group A (50% vs. 26.2%, p = 0.02). This difference was statistically significant. All patients in Group B (100%) and 20 patients in Group A (23.8%) had KDIGO Stage III AKI, with the between-group difference being statistically significant (p < 0.01).

**Table 1:** Baseline demographic, clinical, and laboratory characteristics of patients in the conservative and interventional groups ^*^Indicate statistically significant results (p < 0.05) NO-APN: nonobstructive pyelonephritis; AKI: acute kidney injury; SD: standard deviation; COVID-19: coronavirus disease 2019

Dependent variables	NO-APN with AKI		Test used	Statistic	P-value
	Conservative (n=84)	Interventional (n=24)			
	N (%)	N (%)			
Age group, years, n (%)					
20 – 39	12 (11.9%)	1 (4.2%)	z-test	1.14	0.25
40 – 59	22 (26.2%)	10 (41.7%)		1.42	0.18
>60	52 (61.9%)	13 (54.2%)		0.7	0.48
Age, years, mean ± SD	61.4 ± 16.5	70.4 ± 8.2	t-test (Welch)	3.58	0.01^*^
Sex, n (%)					
Male	53 (63.1%)	17 (70.8%)	Chi-square	0.49	0.46
Female	31 (36.9%)	7 (29.2%)			
Underlying condition, n (%)					
Diabetes mellitus	17 (20.2%)	3 (12.5%)	z-test	0.89	0.37
Hypertension	10 (11.9%)	1 (4.2%)		1.14	0.25
Diabetes with hypertension	40 (47.6%)	14 (58.3%)		0.86	0.38
Cardiovascular disease	12 (14.3%)	13 (54.2%)		4.11	0.01^*^
Significant past history, n (%)					
AKI	6 (7.1%)	3 (12.5%)	z-test	0.79	0.42
Urosepsis	22 (26.2%)	12 (50%)		2.23	0.02^*^
Renal calculi	2 (2.4%)	1 (4.2%)		0.55	0.57
Recurrent UTI	2 (2.4%)	1 (4.2%)		0.55	0.57
COVID-19	6 (7.1%)	2 (8.3%)		0.86	0.86
Symptoms, n (%)					
Fever	54 (64.3%)	16 (66.7%)	z-test	0.18	0.78
Chills/rigor	31 (36.9%)	4 (16.7%)		1.93	0.06
Nausea/vomiting	21 (25.0%)	4 (16.7%)		0.92	0.41
Loin pain	27 (32.1%)	12 (50%)		1.62	0.1
Signs, n (%)					
Tachycardia	15 (17.9%)	4 (16.7%)	z-test	0.11	0.91
Hypotension	4 (4.8%)	2 (8.3%)		0.65	0.71
Temperature, °F, mean ± SD	98.9 ± 1.4	99.3 ± 1.6	t-test (Welch)	1.19	0.23
Laboratory findings, , n (%)					
Leukocytosis	45 (53.6%)	14 (58.3%)	z-test	0.43	0.72
Thrombocytopenia	48 (57.1%)	12 (50%)		0.6	0.54
HbA1c, %, mean ± SD	8.4 ± 1.7	9.3 ± 2.1	t-test (Welch)	2.91	0.03^*^
AKI, n (%)					
Stage 1	5 (6.0%)	0 (0)	z-test	1.22	0.21
Stage 2	59 (70.2%)	0 (0)		6.07	0.01^*^
Stage 3	20 (23.8%)	24 (100%)		6.67	0.01^*^

Table 2 shows organisms isolated from urine and blood cultures. Escherichia coli was the predominant isolate in both groups (Group B 47.1% vs. Group A 38.1%; p = 0.72). Candida species were the second most common (33.3% vs. 31.0%; p = 0.85), followed by Klebsiella species (12.5% vs. 14.3%; p = 0.90). There was no statistically significant difference in the distribution of organisms between the two groups.

**Table 2:** Causative organisms isolated from urine and blood cultures among patients in both treatment groups Comparisons were performed using the z-test for proportions. P-value < 0.05 is considered significant NO-APN: nonobstructive pyelonephritis; AKI: acute kidney injury

Causative organism isolated	NO-APN with AKI		Test used	Statistics	P-value
	Conservative (n=84), n (%)	Interventional (n=24), n (%)			
Urine culture					
Escherichia coli	32 (38.1%)	10 (41.7%)	z-test	0.32	0.72
Enterococcus species	7 (8.3%)	3 (12.5%)		0.62	0.45
Candida species	26 (31.0%)	8 (33.3%)		0.22	0.85
Klebsiella species	12 (14.3%)	3 (12.5%)		0.23	0.90
Gram-negative bacteria	7 (8.3%)	0 (0)		1.46	0.15
Blood culture					
Gram-negative bacteria	31 (36.9%)	11 (45.8%)	z-test	0.79	0.42
Escherichia coli	17 (20.2%)	2 (8.3%)		1.35	0.17
Staphylococcus species	18 (21.4%)	7 (29.2%)		0.79	0.41
Salmonella	3 (3.6%)	4 (16.7%)		2.29	0.11

There was no statistically significant difference between conservative (Group A) and intervention (Group B) patients regarding overall renal outcomes. A subgroup analysis comparing Stage III AKI patients in Group A (n = 20) with Group B (n = 24) was then performed. Intragroup comparison was carried out for admission versus discharge values of serum creatinine and TLC. Group B demonstrated better treatment outcomes than Group A, showing a significant reduction in mean TLC (14,937.9 ± 5,275.5 → 11,656.3 ± 4,936.1 cells/mm³; p = 0.03) and serum creatinine (3.46 ± 2.50 → 2.19 ± 1.93 mg/dL; p = 0.05*). Details are provided in Table 3.

**Table 3:** Paired t-test comparing intra-group outcomes (Stage III AKI) ^*^Indicate statistically significant results (p < 0.05) AKI: acute kidney injury; SD: standard deviation

Subgroup analysis (Stage III AKI)	Mean ± SD		Mean difference	t-statistic	P-value
	During admission	At discharge			
Conservative treatment (n = 20)					
Total leucocyte count, cells/m^3^	12437.5 ± 4917.7	10747.9 ± 3366.9	1689.60	1.27	0.21
Serum creatinine level, mg/dL	3.50 ± 1.53	2.90 ± 2.17	0.59	1.01	0.31
Minimally invasive procedure (n = 24)					
Total leucocyte count, cells/m^3^	14937.9 ± 5275.5	11656.3 ± 4936.1	3281.60	2.25	0.03^*^
Serum Creatinine level mg/dL	3.46 ± 2.50	2.19 ± 1.93	0.64	1.97	0.05^*^

Comparison of hospitalization duration between Stage III AKI patients in Groups A and B revealed no significant difference (7.35 ± 4.28 days vs. 7.13 ± 3.11 days; p = 0.84), as summarised in Table 4.

**Table 4:** Independent t-test comparing duration of hospital stay between conservative and minimally invasive (DJ stenting) groups AKI: acute kidney injury; SD: standard deviation

Subgroup analysis (Stage III AKI)	Mean ± SD		Mean difference	t-statistic	P-value
	Conservative treatment (n=20)	Minimally invasive procedure (n=24)			
Duration of hospitalization, days	7.35 ± 4.28	7.13 ± 3.11	0.230	0.206	0.84

## Discussion

Our study of NO-APN in acute AKI patients showed a high incidence of KDIGO Stage II and Stage III AKI in hospitalized patients, with 54% (n = 59) presenting with or progressing to KDIGO Stage II AKI, while 40% (n = 49) reached Stage III. In contrast, Jeon et al. [1] reported AKI in 62% of APN cases, with 27% progressing to Stage II and 11% to Stage III. Another study from India by Yadla et al. [6] showed a similar incidence of AKI (62%), with 26% and 12% developing Stage II and Stage III AKI, respectively. However, in our study, the majority had significantly Stage II and III AKI. The higher proportion of advanced AKI in our cohort may be attributable to our institution’s role as a tertiary care referral center. This overall increasing incidence of AKI and its severity in NO-APN cannot be attributed to a single cause, but rather to multiple factors amplifying the kidney’s vulnerability to injury. These include the robust inflammatory response triggering cytokine-mediated direct tubular injury, sepsis-mediated hemodynamic instability, resistance to antibiotics, and underlying host factors, including pre-existing kidney damage, diabetes, and anatomic abnormalities.

The mean age of participants was similar in both groups: 61.4 ± 16.5 years vs. 70.4 ± 8.2 years. There was no significant difference in age or sex distribution between the two groups. However, males were more frequently affected than females. The majority of participants had both diabetes and hypertension - 40 (47.6%) in the conservative group vs. 14 (58.3%) in the DJ stent group - as coexisting morbidities. Additionally, a history of prior urinary tract infection (UTI) was higher in Group B. Laboratory findings indicated leukocytosis and thrombocytopenia were equally distributed in both groups, except for a higher mean HbA1c level in the DJ stent group compared to the conservative treatment group, which was statistically significant. These findings align with the observations of Kumar et al. [7], who studied factors influencing the need for DJ stenting in patients with APN. In their study, the intervention group had a higher incidence of previous UTI and higher mean HbA1c levels. Yadla et al. [6] also reported a similar clinical profile in NO-APN patients with AKI, though without correlation to high HbA1c levels.

The key feature of our study was the pre-specified subgroup analysis among patients with Stage III AKI in the conservative group versus the DJ stenting group. This assessment was planned because DJ stenting, as a minimally invasive procedure in NO-APN with AKI, is usually performed in patients with advanced stages of AKI, and all the patients in Group B who underwent DJ stenting had Stage III AKI. In our study, the intervention group demonstrated better treatment outcomes than the conservative treatment during intra-group comparison, implying that DJ stenting had an additional benefit over antibiotics alone in APN patients with Stage III AKI. However, there was no significant difference in the duration of hospitalization between the two groups.

Gundlapalli et al. [8], in their study on the role of stenting in acute pyelonephritis, reported that patients who failed to respond to conservative management were effectively managed by ureteric stenting, and the mean estimated glomerular filtration rate (eGFR) and leukocytosis improved after stenting. They concluded that early ureteric stenting in such patients improves renal outcomes in APN. Similarly, Gite et al. [9] reported that minimally invasive procedures had a positive impact in preserving renal function, especially when medical management fails. The study also recommended that patients with poor prognostic factors be treated aggressively in the acute phase using minimally invasive techniques. Kumar et al. [7] also noted significant improvements in serum creatinine and TLC following minimally invasive procedures, although their study did not specifically address NO-APN.

Our study is the first from India to examine the role of DJ stenting in NO-APN with AKI. The plausible mechanisms underlying the beneficial role of DJ stenting in cases without significant obstruction remain unclear. A certain degree of ureteric dyskinesia has been well documented in APN and is attributed to the effects of bacterial toxins and inflammatory mediators on ureteric smooth muscles. Additionally, purulent debris and flakes, along with necrosed papillae, might contribute to aperistalsis of the ureter, even when not severe enough to produce a radiologically significant obstruction. DJ stenting helps in overcoming the aperistalsis of the ureter, draining away the purulent urine, indirectly contributing to local sepsis control and thereby aiding renal recovery.

Limitations and strengths

This study has certain limitations. It involves a retrospective data analysis, and a prospective randomized controlled trial would be required to validate these findings. Additionally, the smaller sample size in Group B, along with higher baseline HbA1c levels and a greater history of prior urosepsis episodes in this group, may have influenced the results. Nevertheless, the pre-specified subgroup analysis allowed for a direct comparison between the intervention and conservative groups, thereby overcoming the effect of the above confounding factors. Though the study is not powered to strongly recommend the use of DJ stenting as an adjunct therapy in all cases of NO-APN with AKI, it does emphasize its potential benefit in patients with more advanced stages of AKI.

## Conclusions

Our study provides significant insights into the treatment of NO-APN patients with severe AKI. The magnitude and severity of the disease are evident from the fact that more than 40% of our cohort developed KDIGO Stage III AKI. The subgroup analysis of stage III AKI patients between the two arms showed a significant beneficial effect of adding DJ stenting to standard parenteral antibiotic therapy. While this approach enhanced renal recovery and sepsis control, it did not make a difference in the duration of hospital stay. These findings are noteworthy, given that the current standard of care remains predominantly conservative, employing parenteral antibiotics.
